# Virtual screening identifies specnuezhenide as a potential therapeutic agent for rosacea via KLK5/TLR4/NF-κB pathway inhibition and metabolic modulation

**DOI:** 10.3389/fimmu.2026.1886252

**Published:** 2026-07-29

**Authors:** Jingang Xu, Xinyu Li, Manyu Chen, Shahong Di, Yao Zhang, Yueye Xu, Boya Xu, Junjie Guo, Jingyun Xu, Yuanyuan Li, Jinhong Zhao

**Affiliations:** 1 Department of Medical Parasitology, Wannan Medical University, Wuhu, Anhui, China; 2 Department of Clinical Pathology, The First Affiliated Hospital of Zhejiang University School of Medicine Suzhou Branch, Suzhou, Anhui, China; 3 School of Basic Medical Sciences, Wannan Medical University, Wuhu, Anhui, China; 4 School of Public Health, Wannan Medical University, Wuhu, Anhui, China; 5 Department of Medical Parasitology, Qiqihaer Medical College, Qiqihaer, Heilongjiang, China; 6 Anhui Provincial Key Laboratory of Biological Macro-molecules, Wuhu, Anhui, China

**Keywords:** KLK5, metabolomics, rosacea, specnuezhenide, virtual screening

## Abstract

**Background:**

Rosacea is a chronic inflammatory skin condition. Excessive LL-37 is produced when serine protease kallikrein-5 (KLK5) is hyperactivated, contributing to the inflammatory response in rosacea. Thus, inhibition of KLK5 has the potential to attenuate inflammation in this condition. Reports suggest that specnuezhenide (Spe), an isomer of oleanolic acid, is effective as an anti-inflammatory agent for the treatment of inflammatory diseases. However, little is known about the role of Spe and its mechanisms in alleviating rosacea.

**Methods:**

Spe was identified as a potential KLK5 inhibitor through virtual screening and molecular docking, showing high-affinity binding via hydrophobic and hydrogen-bond interactions. Rosacea was induced in mice by intradermal LL-37 injection, followed by intraperitoneal administration of Spe. The severity of skin inflammation was assessed histologically. Serum cytokines, inflammatory gene expression, and protein levels of CD31, MMP-9, CD4, and KLK5 were analyzed by ELISA, RT-qPCR, and immunofluorescence, respectively. Inhibition of the TLR4/NF-κB pathway was examined by Western blot. Untargeted metabolomics was applied to characterize alterations in serum metabolites.

**Results:**

Spe markedly reduced LL-37-induced skin inflammation, decreased inflammatory cell and mast cell infiltration, decreased MMP-9 expression, and reduced CD31 fluorescence intensity and CD4^+^ T cell recruitment. The study demonstrated that Spe inhibited the expression of pro-inflammatory cytokines (IL-1β, IL-6, and TNF-α) in serum and suppressed activation of TLR2, MMP-9, KLK5, IL-1β, IL-6, and TNF-α genes. Moreover, Spe inhibited KLK5 expression and blocked activation of the TLR4/NF-κB pathway. Metabolomics identified 43 dysregulated metabolites in rosacea, 26 of which were normalized by Spe treatment. Pathway enrichment analysis suggested that regulation of unsaturated fatty acid biosynthesis is a key mechanism underlying the effects of Spe.

**Conclusions:**

This study demonstrates for the first time that Spe, a novel KLK5 inhibitor, suppresses the inflammatory response mediated by the KLK5/TLR4/NF-κB pathway, accompanied by metabolic remodeling, particularly involving the biosynthesis of unsaturated fatty acids, thereby ameliorating rosacea-like dermatitis.

## 1 Introduction

Rosacea is a chronic dermatologic condition characterized by flushing, erythema, telangiectasia, papules, and pustules (1). Epidemiologic studies report a prevalence ranging from 2% to 22% of the population (2). Erythema, papules, and pustules accompanied by facial redness are common symptoms and often contribute to low self-esteem and depression, which may impair social interactions (3). In the pathophysiology of rosacea, recent research has emphasized the roles of metabolic dysfunction, neuroimmune interactions, microbial dysbiosis, and dysregulation of the sebaceous glands (4). Current treatment strategies primarily focus on symptom management using metronidazole, azelaic acid, and ivermectin; however, these provide limited long-term disease control (1). Therefore, the development of novel therapeutic strategies remains a priority. Serine protease kallikrein-5 (KLK5) plays a central role in cleaving cathelicidin antimicrobial peptides (CAMPs) to generate LL-37, an active peptide that drives inflammation in rosacea (5). KLK5 is crucial for the activation of inactive cathelicidin into LL-37, and elevated LL-37 levels in lesional skin are indicative of high KLK5 activity (6). The FDA-approved medications commonly utilized in the treatment of inflammatory rosacea are oral doxycycline and azelaic acid (7). Topical azelaic acid (AZA) and oral doxycycline can reduce papulopustular lesions and perilesional erythema in rosacea, activng via direct and indirect inhibition of KLK5 activity, respectively. (5, 8) Inhibition of KLK5 has been associated with reduced papules and erythema, providing further evidence of its role in rosacea pathophysiology (9). Thus, agents capable of inhibiting KLK5 production or activity are expected to suppress LL-37 expression and attenuate the inflammatory cascade associated with rosacea (10).

Virtual screening (VS) provides a cost-effective approach to rapidly identify novel hit compounds (11). Advances in computational modeling have further enhanced the utility of VS in drug discovery (12). The success of FDA-approved drugs identified through VS and molecular docking underscores the value of these approaches (13).

Specnuezhenide (Spe), an isomer of oleanolic acid, is commonly found in herbal medicine and nature. It has demonstrated antioxidant, anti-inflammatory, and hypoglycemic effects (14). Previous studies have demonstrated its protective effects against kidney injury through suppression of inflammatory mediators such as TNF-α, IL-6, and IL-1β (15). Spe has also been reported to ameliorate osteoarthritis in rats via inhibition of NF-κB and Wnt/β-catenin pathways (16). In diabetic rats, Spe suppressed NF-κB expression, reducing vascular inflammation (17). However, whether Spe improves rosacea-like lesions remains unknown.

Metabolomics, a rapidly evolving discipline, employs mass spectrometry to monitor and identify metabolites, thereby revealing their metabolic roles (18). It has been widely applied in drug target screening, disease diagnosis, and personalized therapy, particularly by analyzing differences in metabolites between experimental and control groups to uncover underlying physiological and pathological pathways (19). Non-targeted metabolomics enables the assessment of diverse endogenous and exogenous metabolites in small biological samples. Linking altered metabolites to biological pathways can provide insight into mechanisms of action *in vivo*, offering precise therapeutic targets (20). For instance, one study reported a distinctly altered metabolic profile in rosacea patients, especially involving amino acid-related pathways (21). Similarly, Yan (22) examined immune-mediated inflammatory disorders through metabolomics. Nonetheless, only a limited number of metabolomic studies have been performed in rosacea mouse models.

The present study aimed to identify novel KLK5 inhibitors through virtual screening and to investigate whether Spe, as a potent KLK5 inhibitor, exerts therapeutic effects on LL-37-induced rosacea-like lesions in mice, while also exploring the potential mechanisms involved.

## 2 Materials and methods

### 2.1 Structure-based virtual screening

All virtual screening (VS) procedures were performed using the Yinfo Cloud Platform (https://cloud.yinfotek.com/). The prepared L6020, L6030, and Targetmol libraries were used for VS. The L6020 and L6030 libraries were initially screened using AutoDock Vina (23), and the top 1,000 compounds were selected. These compounds were further screened with DOCK 6.9 (24). The Targetmol library was directly screened with DOCK 6.9. The crystal structure of the Kallikrein-5 protein (Protein Data Bank ID: 6QFE) was obtained from the RCSB Protein Data Bank database (http://www.rcsb.org/) using the platform widget [Processing PDB Structures]. AutoDock Tools was used to assign AD4 atom types with Kollman charges to the protein structure. Semi-flexible docking was then performed with AutoDock Vina to output the best conformation for each compound. The second round of molecular docking was carried out with the DOCK 6.9 protocol, generating 10,000 conformational orientations per compound, which were scored using Grid for binding evaluation. Candidate conformations were analyzed by clustering, and the best-scored conformation was selected. Compounds with a Grid Score > –70 kcal/mol were excluded. The details of the integration are as follows:

1. Binding pocket definition: The binding pocket was defined based on the crystal structure of KLK5 (PDB ID: 6QFE), centered around the catalytic triad residues His57, Asp102, and Ser195, with a radius of 10 Å encompassing all residues within the active-site cleft.
2. Docking box dimensions: The docking grid box was centered on the catalytic triad and set to dimensions of 30 × 30 × 30 Å³ (grid spacing: 0.375 Å), which was sufficiently large to accommodate both the binding site and ligand flexibility.
3. Validation of the docking protocol: To validate the reliability of our docking protocol, we performed a redocking experiment using the co-crystallized ligand. The RMSD between the redocked pose and the crystallographic conformation was 0.93 Å (< 2.0 Å), confirming that our docking parameters could successfully reproduce the experimental binding mode.
4. Positive control ligand: The co-crystallized inhibitor J08 (GSK144), a known KLK5 inhibitor with a reported Half Maximal Inhibitory Concentration of 0.08 μM, was included as a positive control for binding affinity comparison.
5. Docking accuracy metrics: Docking accuracy was evaluated using RMSD (< 2.0 Å), Grid_Score, Grid_vdw, and Grid_es for binding energy estimation (as presented in **Table 1**). Z-score normalization was also applied to docking scores across the compound library to ensure comparability.

**Table 1:** Molecular docking scores of KLK5 with Spe (kcal/mol).

Compounds	Grid_score	Grid_vdw	Grid_es	Int_energy
449733-84-0	-117.28	-108.04	-9.24	18.84

The final seed compound, Spe, was manually analyzed, and its binding mode was visualized using PyMOL mapping. Moreover, this study employed Gromacs 2024.2 to conduct molecular dynamics simulations of KLK5-Spe. The small molecule underwent preprocessing with sobtop, incorporating the GAFF force field and charges. Simulations were performed at 300 K and 1 bar, using the Amber03 force field and Tip3p water model, with the system charge neutralized by Na^+^. The simulation sequence comprised energy minimization (using the steepest descent method), 500 ps each of NVT and NPT equilibration (coupling constant 0.1 ps), followed by 100 ns of free dynamics simulation (2 fs step size). Structural stability, flexibility, and solvent accessibility of the complex were evaluated by analyzing parameters including RMSD, RMSF, Rg, and SASA.

### 2.2 Materials

LL-37 was purchased from MedChemExpress (New Jersey, USA). Doxycycline hydrochloride was obtained from Shanghai Macklin Biochemical Technology Co., Ltd. (Shanghai, China). Specnuezhenide and KLK5 were purchased from Shanghai TargetMol Biotech Co., Ltd. (Shanghai, China). Primary antibodies included the following: Anti-TLR4 (Rabbit mAb, Cat. No. #14358, CST), Anti-p65 (RelA) (Rabbit mAb, Cat. No. #8242, CST), and Anti-phospho-p65 (Ser536) (Rabbit mAb, Cat. No. #3033, CST). Goat Anti-Rabbit IgG was purchased from Beijing Labgic Technology Co., Ltd. (Haimen, China).

### 2.3 Animals and treatment

A total of 49 6–8-week-old Specific Pathogen Free BALB/c female mice were purchased from Henan Skebes Bio-technology Co., Ltd. (Production License No. SCXK(Yu)2020-0005). The mice were housed under SPF conditions at the Laboratory Animal Center of Wannan Medical College, maintained at 24 °C ± 2°C, with free access to food and water. Experiments began after one week of acclimatization. All procedures were approved by the Animal Ethics Committee of Wannan Medical College (Approval No. SUCMDL20220805002). Spe was dissolved in a mixed vehicle consisting of 10% DMSO, 40% PEG300, 5% Tween 80, and 45% saline, while doxycycline hydrochloride (DH) was dissolved in saline.

The mice were randomly divided into seven groups (n = 7 mice per group): control (PBS), model (LL-37), doxycycline hydrochloride (DH, 30 mg/kg/d), LL-37 + low-dose Spe (20 mg/kg/d), LL-37 + medium-dose Spe (40 mg/kg/d), LL-37 + high-dose Spe (60 mg/kg/d), and LL-37 + high-dose Spe + KLK5 (60 mg/kg/d + 2 μg KLK5). Group sizes (n = 7) were determined based on efficacy analysis and comparable prior studies, ensuring adequate statistical power to detect treatment effects at the 5% significance level with 80% efficacy.

**Figure 1:**
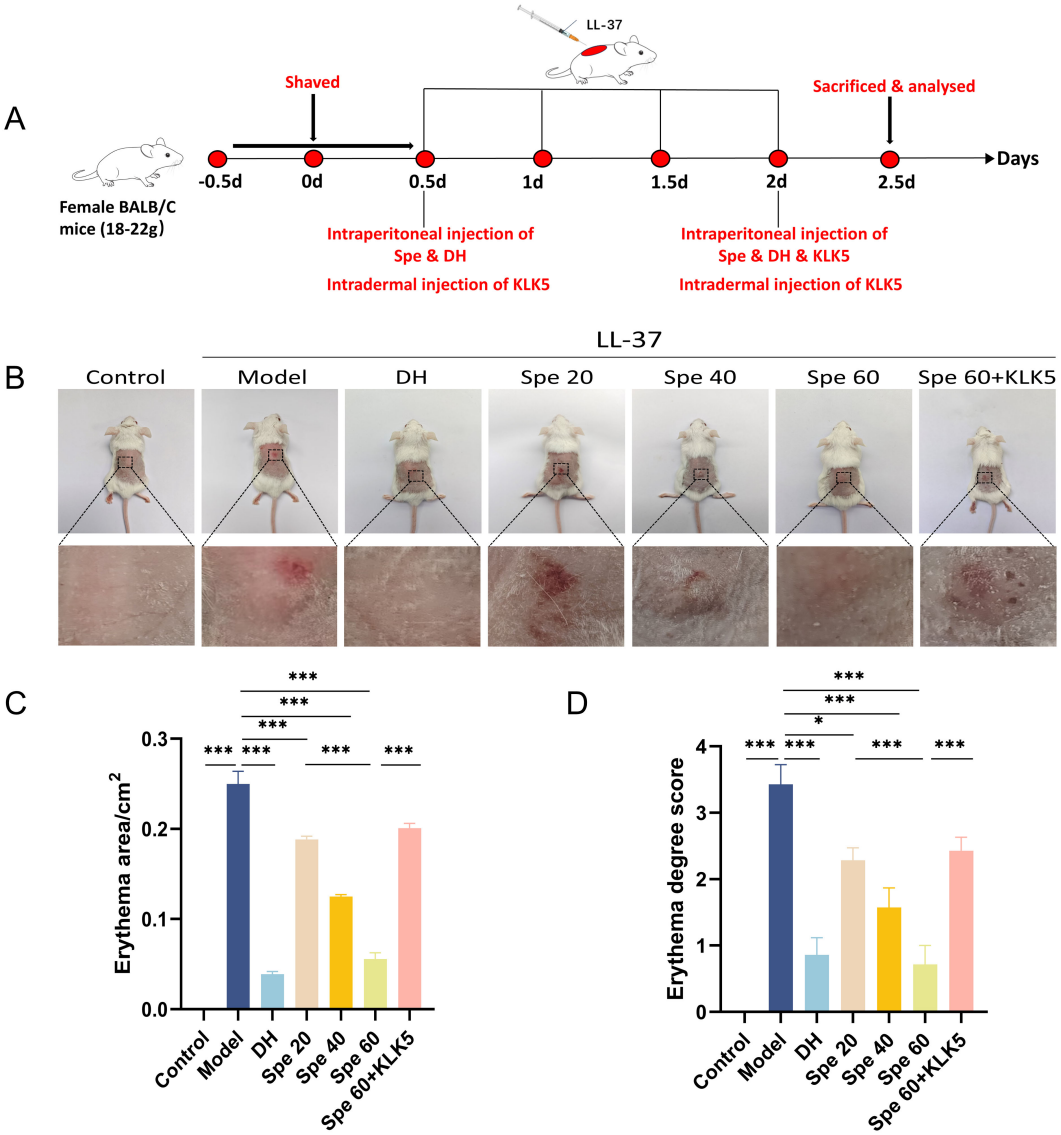
Effects of Spe on rosacea-like skin lesions in LL-37-induced mice at 2.5 days after treatment. **(A)** Schematic diagram of mouse modeling, drug administration, and skin measurements; **(B)** gross photographs representative of seven mice; **(C)** erythema area of seven mice in different groups; and **(D)** erythema score of seven mice in different groups. Data are presented as mean ± SEM, *p < 0.05, ***p < 0.001. DH, doxycycline hydrochloride.

All mice were shaved 24 h prior to treatment (a 3 × 3 cm area). Mice in the rosacea model and Spe-treated groups were injected subcutaneously with 40 μL of LL-37 (320 μM) into the dorsal skin twice daily for two consecutive days, as previously described. Control mice received 40 μL PBS subcutaneously. The doxycycline group received intraperitoneal injections of 30 mg/kg/d, administered every 24 h beginning 12 h after LL-37 injection, for a total of two injections. Spe was administered intraperitoneally at 20, 40, or 60 mg/kg/d for two consecutive days. For the Spe + KLK5 group, KLK5 (2 μg) was injected into the dorsal skin every 24 h for two injections (**Figure 1A**). Inflammation severity was evaluated 12 h after the last LL-37 injection by assessing erythema, as previously described (25).

### 2.4 Evaluation of skin inflammation severity

Dorsal skin lesions were photographed 12 h after the final injection, and rosacea-like severity was scored based on erythema intensity and area (26). Erythema severity was graded from 0 to 4 as follows: 0, no erythema; 1, faint erythema; 2, mild erythema; 3, obvious and well-defined erythema; and 4, dark erythema with clear borders. The erythema scores were assessed by two independent investigators who were unaware of the group allocation.

### 2.5 Histopathological analysis

No anesthetic was used; animals were directly euthanized by an intraperitoneal overdose of sodium pentobarbital (150–200 mg/kg), which induces rapid loss of consciousness followed by cardiorespiratory arrest. After euthanasia, dorsal skin samples were collected and fixed in 10% neutral buffered formalin. Samples were dehydrated with graded ethanol, cleared with xylene, and embedded in paraffin (27). Sections (4 μm) were prepared and stained with hematoxylin and eosin (H&E) and toluidine blue (TB). A light microscope (Olympus BX53, Japan) with a digital camera (Olympus DP22, Japan) was used to analyze the slides at 200× and 400× magnifications. Using ImageJ software (NIH, USA), the numbers of mast cells and inflammatory cells in the dermis were measured from five randomly chosen fields on three slides.

### 2.6 Immunofluorescence

After fixing skin tissues in 4% paraformaldehyde (PFA) for 24 h at 4 °C, they were rinsed with PBS and then incubated with 15% sucrose in PBS for another 24 h at 4 °C until the tissues sank. Samples were then prepared into 8 μm cryostat sections after being embedded in optimal cutting temperature (OCT) compound. Following a 20-min air-drying period at room temperature, the sections were cleaned with PBS and blocked using 5% BSA and 0.1% Triton X-100 in PBS. After being treated with primary antibodies overnight at 4 °C, the sections were rinsed with PBS and incubated for 1 h with secondary antibodies. A medium containing DAPI (Abcam) was used to mount the slides (28). The following major antibodies were employed: Rat anti-KLK5 (1:100, eBioscience), Rat anti-MMP-9 (1:100, eBioscience), Rat anti-CD4 (1:100, eBioscience), and Rat anti-CD31 (1:100, eBioscience).

### 2.7 ELISA assay

Blood was collected via orbital venous plexus puncture immediately after euthanasia, and serum was separated by centrifugation at 3,000 rpm for 15 min at 4 °C and stored at –80 °C. Serum concentrations of IL-1β, IL-6, and TNF-α were determined using ELISA (enzyme-linked immunosorbent assay) kits (Ruixin Biological Company, Quanzhou, China) according to the manufacturer’s instructions. Specifically, 50 μL of each serum sample was added to 96-well plates pre-coated with monoclonal antibodies specific for mouse TNF-α, IL-1β, and IL-6. After incubation in a thermostat at 37 °C for 1 h in the dark, the plates were washed, and horseradish peroxidase (HRP)-labeled detection antibodies were applied. After the plates were washed five times with PBS containing 0.05% Tween-20, substrates A and B were added. The reaction was then incubated for 15 min at 37 °C in a thermostat in the dark. A halting solution was added to terminate the reaction. Using an ELISA microplate reader (Molecular Devices, USA), absorbance was measured at 450 nm.

### 2.8 RT-qPCR assay

Trizol reagent (ShareBio) was used to extract total RNA from dorsal skin tissue, and the manufacturer’s instructions were followed to measure RNA concentration. Taq DNA polymerase was then used for amplification after total RNA was reverse-transcribed into cDNA. Using an Applied Biosystems (Roche LightCycler 96) heat cycler, 40 cycles of PCR amplification were carried out, with 30 s of denaturation at 95 °C and 30 s of annealing at 60 °C. Melting curves were generated, and the 2-ΔΔCT technique was used to evaluate the data. The internal reference gene was GAPDH. Target gene sequences were obtained from the National Center for Biotechnology Information (NCBI) database, and Primer5 software was used to create primers. Bioengineering (Shanghai) Biological Engineering Co., Ltd. synthesized the primers. The sequences are shown in **Table 2**.

**Table 2:** Forward and reverse primer sequences for GAPDH, IL-1β, IL-6, TNF-α, TLR2, MMP-9, and KLK5.

Gene	Primers	Sequence (5’-3’)
GAPDH	Forward	ACCACAGTCCATGCCATCAC
	Reverse	GTGAGGGAGATGCTCAGTGT
IL-1β	Forward	AAAAAAGCCTCGTGCTGTCG
	Reverse	GTCGTTGCTTGGTTCTCCTTG
IL-6	Forward	TGATGGATGCTACCAAACTGGA
	Reverse	TGTGACTCCAGCTTATCTCTTGG
TNF-α	Forward	CAGGCGGTGCCTATGTCTC
	Reverse	CGATCACCCCGAAGTTCAGTAG
TLR2	Forward	TCTAAAGTCGATCCGCGACAT
	Reverse	CTACGGGCAGTGGTGAAAACT
MMP-9	Forward	GGTCCTCACCATGAGTCCCT
	Reverse	AGACCACAAAAGTCGGCTGG
KLK5	Forward	CAGAACCACTTAGCCTCGAC
	Reverse	AGGGTAGCCATTGCCCATTT

### 2.9 Western blot assay

The expression levels of TLR4, p65, and pp65 proteins were analyzed by Western blot. Skin tissues were rapidly frozen in liquid nitrogen and stored at –80 °C. Samples were homogenized with an ultrasonic processor, and proteins were extracted using RIPA lysis buffer. After centrifuging the lysates for 20 min at 4 °C at 12,000 rpm, the supernatant was collected. A bicinchoninic acid (BCA) test kit was used to assess the amount of protein present. Equal quantities of protein were separated by 10% SDS-PAGE and then transferred to PVDF membranes (Beyotime, China). Primary antibodies (1:1000 dilution) were used to block the membranes and incubate them overnight at 4 °C. HRP-conjugated secondary antibodies were administered for 1 h the next day. A femto-type ECL reagent (ShareBio) was used to visualize immunoreactive bands, while GAPDH served as the internal control. In order to ensure reproducibility, each experiment was conducted three times.

### 2.10 Metabolomics

Serum samples were analyzed for metabolomic profiling using liquid chromatography–mass spectrometry (LC-MS). Samples were stored at –80 °C, thawed on ice, vortexed, and centrifuged at 12,000 ×g for 1 min at 4 °C. Quality control samples (PQC) and system suitability test samples (SST) were prepared. Metabolite extraction was performed using pre-cooled methanol (–80 °C) as the extraction solvent. After the addition of internal standard solution, the mixture was vortexed, shaken, and incubated at –20 °C for 30 min, then centrifuged at 14,000 ×g for 10 min at 4 °C. The supernatant was freeze-dried and reconstituted in 10% aqueous methanol for LC-MS analysis. Using a Waters ACQUITY BEH C18 column (1.7 µm × 2.1 mm × 100 mm), chromatographic separation was carried out. Two mobile phase systems were used: (1) 0.1% formic acid aqueous solution and 0.1% formic acid in acetonitrile/methanol (40/60) for positive and negative ion collection; and (2) 6.5 mM ammonium bicarbonate aqueous solution and 6.5 mM ammonium bicarbonate methanol solution for negative ion collection. Mass spectrometry was performed using a Q Exactive HF-X spectrometer with a resolution of 60,000 and a scan range of 60–900 m/z.

For non-targeted serum metabolomics, differential metabolites were identified using one-way statistical tests (t-test/Wilcoxon test) combined with multivariate analyses (Variable Importance in Projection (VIP) values from the OPLS-DA model). Screening criteria were set at p < 0.05 and VIP > 1. In OPLS-DA, R2X and R2Y indicate the explanatory power of the X and Y variables, while Q2 reflects predictive accuracy, indicating the fit of the model to real data. All OPLS-DA models were validated by permutation tests (200 times) to assess overfitting and model robustness. Significantly enriched metabolic pathways were identified by KEGG enrichment analysis and metabolite set enrichment analysis (MSEA). MSEA was conducted using the corto package (parameter: 500 substitutions) to assess metabolite set enrichment. Metabolic regulatory networks were visualized using the ggraph package. All analyses were performed in R (version 4.3.2) using the packages ropls (1.34.0), corto (1.2.4), and ggplot2 (3.4.4).

### 2.11 Statistical analysis

GraphPad Prism 8.0 (GraphPad Software, Inc., San Diego, CA, USA) was used for statistical analyses. ImageJ software (National Institutes of Health, USA) was used for image quantification. Data are expressed as mean ± standard error of the mean (SEM). Normality was evaluated using the Shapiro–Wilk test. When dealing with normally distributed data, the Student’s t-test was used to compare two groups, and one-way ANOVA was used to analyze multiple groups. The Kruskal–Wallis test was used for data that were not regularly distributed. Depending on the kind of distribution, Pearson or Spearman correlation coefficients were used to evaluate relationships between numerical variables. The threshold for statistical significance was set at p < 0.05.

## 3 Results

### 3.1 Virtual screening

**Figure 2:**
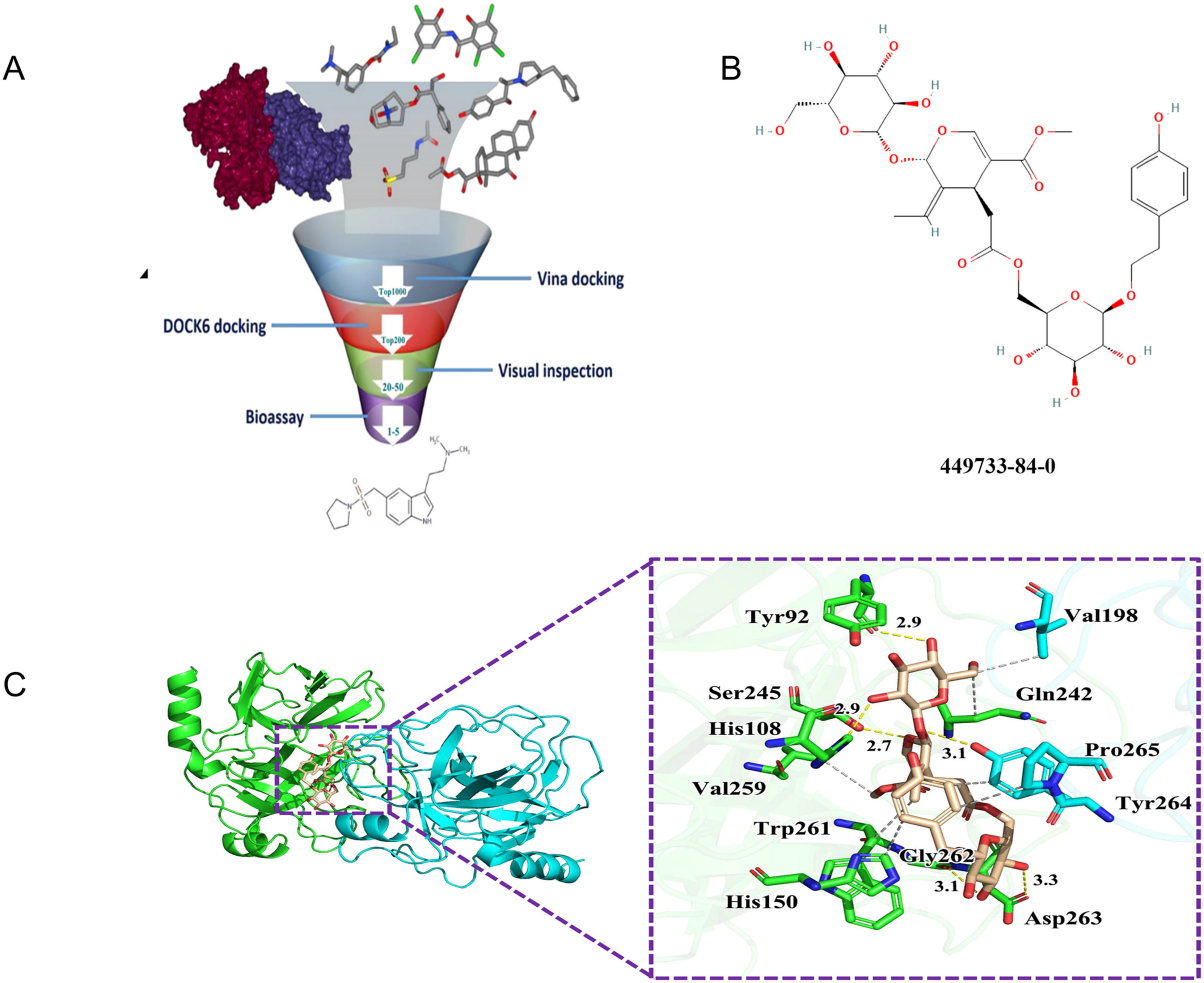
Flowchart for the discovery of Spe, a novel KLK5 inhibitor. **(A)** Virtual screening flowchart; **(B)** chemical structure of Spe (449733-84-0); **(C) b**inding mode of Spe to KLK5. KLK5 A- and B-chains are shown as green and cyan cartoons, respectively; key residues are represented as green and cyan sticks; Spe is shown as wheat-colored sticks. Yellow dashed lines represent hydrogen bonds, and gray dashed lines represent hydrophobic interactions.

Initial screening was performed using VINA (Virtual Screening Tool), followed by rescreening with DOCK 6.9 (Docking and Scoring Function) (**Figure 2A**). Manual analysis of the binding modes of compounds with a Grid Score less than –70 kcal/mol yielded 50 seedling compounds. After preliminary experimentation, specnuezhenide (Spe, 449733-84-0) was identified as capable of binding to KLK5 and inhibiting KLK5 protein activity (**Figure 2B**). The seedling compound Spe was scored and analyzed, and the results of the scoring analysis (**Table 1**) demonstrated that this compound exhibited strong binding to the protein, as indicated by Grid_Score values below –70 kcal/mol, a threshold typically considered indicative of robust binding. This enhanced binding was attributed to the relatively large size of the compound, which enabled the formation of additional hydrophobic interactions within the protein. The overall binding affinity was determined by van der Waals contributions (Grid_vdw) and electrostatic interactions (Grid_es), with van der Waals forces identified as the predominant factor. Nevertheless, electrostatic contributions were also significant, reflecting the presence of multiple polar interactions.

As illustrated in **Figure 2C**, the binding pattern of Spe (449733-84-0) to KLK5 was characterized by interactions with two peptide chains of the protein, predominantly involving residues located in the random coil regions of the secondary structure. These interactions were mainly mediated by hydrophobic contacts and hydrogen bonding, contributing to structural stability. Specifically, hydrophobic interactions occurred between the side chains of Val259 and Trp261 on the A-chain and Tyr264 on the B-chain, and the benzene ring and methyl groups of Spe, resulting in strong van der Waals contributions (Grid_vdw = –108.04 kcal/mol). In addition, hydroxyl groups of Spe formed hydrogen bonds with residues Tyr92, His108, Ser245, Gly262, and Asp263 on the A-chain and Tyr264 on the B-chain, with bond lengths ranging from 2.7 to 3.3 Å, further stabilizing the binding interface (Grid_HBA = –108.04 kcal/mol). These hydrogen bonds served as stabilizing anchors, facilitating small molecule–protein interactions. The electrostatic contributions (Grid_es = –9.24 kcal/mol) also played a critical role in the binding process. The combined action of hydrophobic interactions and hydrogen bonding promoted strong small molecule–protein binding (Grid Score = –117.28 kcal/mol), effectively interfering with protein function. In this molecular dynamics simulation, the root mean square deviation (RMSD), radius of gyration (Rg), residue-wise root mean square free energy (RMSF), and mean square area of surface area (SASA) results for KLK5-Spe are as follows: as shown in Figures D–G, the RMSD of KLK5-Spe was 0.401 nm, indicating stability throughout the simulation with minimal disturbance to the overall protein conformation; regarding Rg, the mean value for KLK5-Spe was 2.539 nm, further indicating no significant alteration in the system’s overall compactness; RMSF analysis revealed a mean value of 0.225 nm for KLK5-Spe, suggesting higher local stability within the system; and SASA data showed a mean value of 221.886 nm² for KLK5-Spe, indicating that the complex formed a tightly packed structure with a small exposed surface area.

### 3.2 Spe dose escalation study reveals its inhibitory effect on LL-37-induced phenotype in rosacea-like mice

Spe was administered at three dose levels, namely, 20 mg/kg/d (low, L), 40 mg/kg/d (medium, M), and 60 mg/kg/d (high, H), to determine the optimal dose for treatment of rosacea-like lesions in mice. Skin erythema severity and area were assessed following treatment. In the LL-37 + H group, mild erythema was observed in 14% (1/7) of mice. In the LL-37 + M group, 71% (4/7) of mice exhibited mild to moderate erythema, while 86% (6/7) of mice in the LL-37 + L group developed mild to moderate erythema. In the doxycycline hydrochloride (DH) group, mild erythema occurred in 14% (1/7) of mice. The average erythema score and area were significantly lower in the Spe 60 group compared with the Spe 20 group (p < 0.001), indicating that treatment with 60 mg/kg/d Spe was effective in alleviating erythema in rosacea-like mice.

A rosacea mouse model was established using LL-37 as described in a previous study (29) to evaluate the therapeutic effects of Spe. In the LL-37-induced model group, marked erythema, inflammatory cell infiltration, and upregulated KLK5/CD4/MMP-9/CD31 expression were observed, confirming the successful establishment of rosacea-like dermatitis. As shown in **Figure 1B**, both Spe and DH effectively reduced erythema induced by LL-37. The erythema score and affected area were significantly decreased in rosacea-like mice treated with Spe (p < 0.05 or p < 0.001). To further examine the role of KLK5 in Spe-mediated improvement, mice were treated with Spe 60 and subsequently administered subcutaneous KLK5 injections. Results demonstrated that mice in the Spe 60 + KLK5 group developed exacerbated dorsal skin erythema, with higher erythema scores and larger erythema areas compared with those in the Spe 60 group (p < 0.001) (**Figures 1C, D**). Collectively, these findings indicate that Spe treatment effectively improves skin phenotype in rosacea-like mice.

### 3.3 Spe inhibits LL-37-induced pathological damage in rosacea-like mice

**Figure 3:**
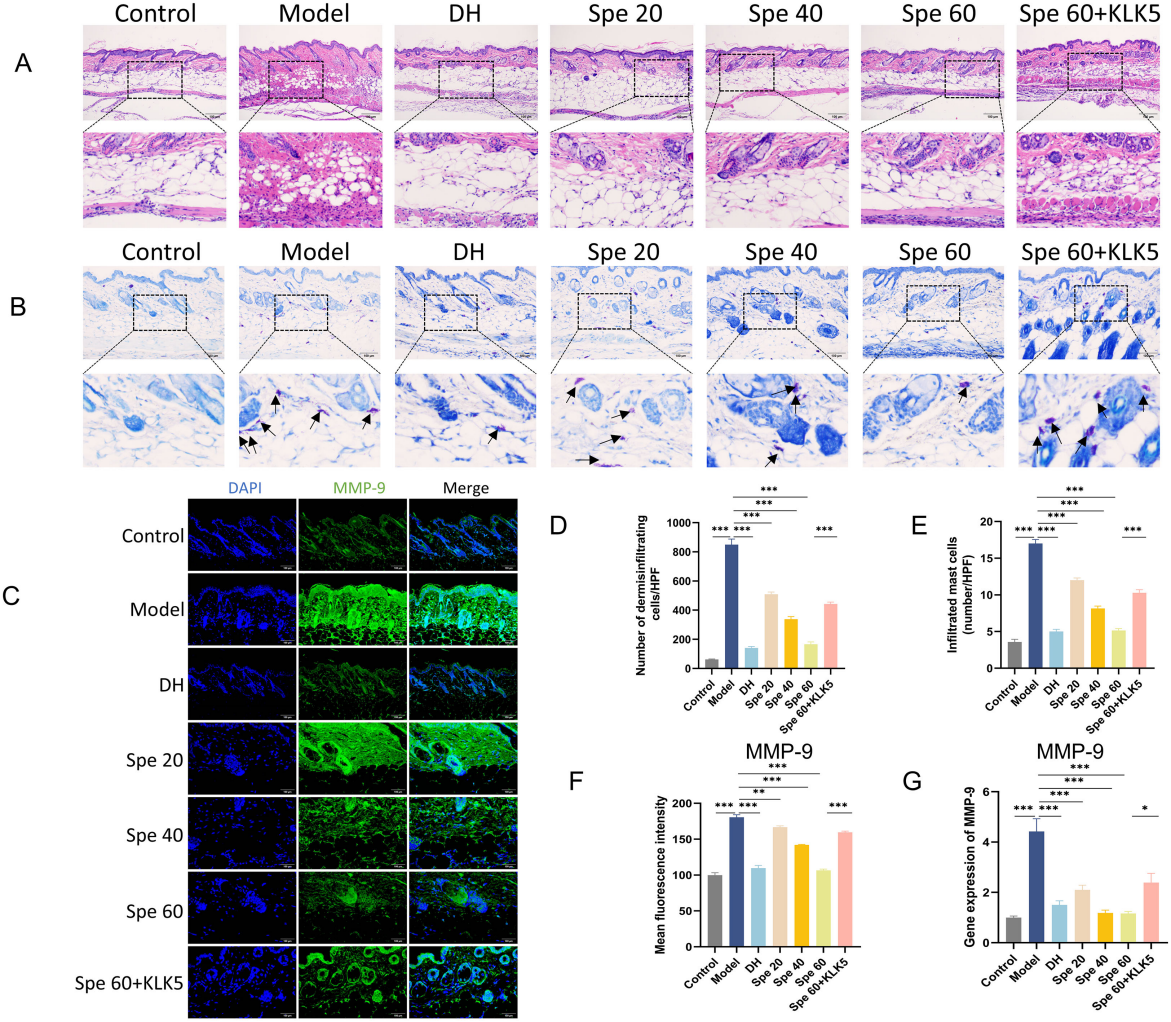
Effects of Spe on pathological features of rosacea-like mouse skin. **(A)** H&E staining for histological analysis of rosacea-like lesions (magnification, 400×; scale bar, 100 μm); **(B)** toluidine blue (TB) staining, black arrows indicate mast cells (magnification, 400×; scale bar, 100 μm); **(C)** immunostaining of MMP-9 in skin lesions from control mice and LL-37-induced mice (scale bar, 50 μm); **(D)** quantification of inflammatory cells; **(E)** mast cell counts from TB-stained sections; **(F)** quantification of dermal MMP-9 fluorescence intensity from control and LL-37-induced mice treated with PBS or Spe (n = 7 per group); and **(G)** expression levels of MMP-9 determined by RT-qPCR. Data are presented as mean ± SEM, *p < 0.05, ***p < 0.001. MMP-9, matrix metallopeptidase 9.

Because inflammatory cells are known to contribute to the pathogenesis of rosacea (30), we investigated whether Spe could alter inflammatory infiltration in rosacea. Mice injected with LL-37 exhibited substantial inflammatory cell accumulation, whereas LL-37-induced mice treated with intraperitoneal Spe displayed markedly reduced inflammatory infiltration in a dose-dependent manner. In contrast, the Spe 60 + KLK5 group showed significantly increased inflammatory cell infiltration and more severe histological skin damage compared with the Spe 60 group (p < 0.001) (**Figures 3A, D**). These findings indicate that Spe alleviates rosacea-like lesions by suppressing inflammatory cell infiltration.

### 3.4 Spe ameliorates pathology in rosacea-like mice by modulating mast cells

Mast cells have attracted increasing attention in the pathophysiology of rosacea due to their ability to activate and release diverse immune mediators. Several studies have reported marked mast cell infiltration in skin lesions of patients with rosacea and in LL-37-induced mice (31). Using toluidine blue staining, we observed that Spe treatment reduced mast cell infiltration in a dose-dependent manner compared with the model group (**Figures 3B, E**).

Activated mast cells contribute to inflammatory amplification by expressing MMP-9, which promotes LL-37 production (32). Therefore, MMP-9 expression was assessed by immunofluorescence. Results showed significantly increased mean fluorescence intensity of MMP-9 in the model group compared with controls (p < 0.001). Spe treatment significantly reduced MMP-9 protein expression in a dose-dependent manner compared with the model group (p < 0.001) (**Figures 3C, F**). RT-qPCR further confirmed these findings, showing elevated MMP-9 mRNA levels in the model group relative to controls, and a dose-dependent reduction in MMP-9 expression following Spe treatment (**Figure 3G**).

Histopathological staining also revealed increased mast cell infiltration in the Spe 60 + KLK5 group compared with the Spe 60 group (p < 0.001). Immunofluorescence staining and RT-qPCR results showed correspondingly higher expression of MMP-9 protein and mRNA in the Spe 60 + KLK5 group (p < 0.05 or p < 0.001). These results indicate that KLK5 reversed the protective effect of Spe 60, exacerbating rosacea-like inflammation. Collectively, these findings suggest that Spe attenuates rosacea-like pathology in mice by modulating mast cell infiltration in a dose-dependent manner.

### 3.5 Spe reduces LL-37-induced skin angiogenesis in rosacea mice

**Figure 4:**
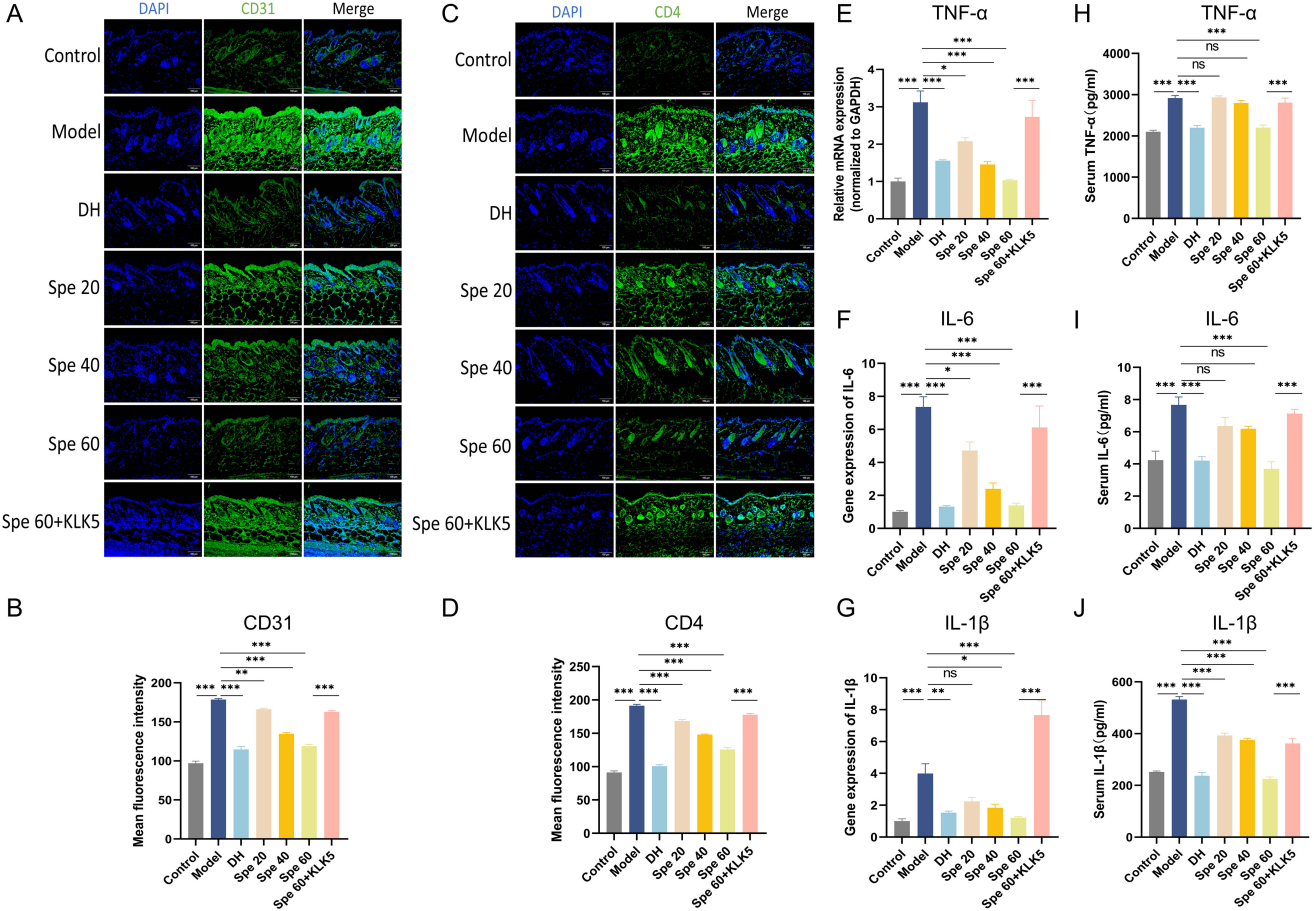
Effects of Spe on angiogenesis and CD4^+^ T-cell infiltration in rosacea-like mouse skin. **(A)** CD31 immunostaining of skin lesions from control and LL-37-induced mice (scale bar, 50 μm); **(B)** quantification of dermal CD31 fluorescence intensity in mice treated with PBS or Spe (n = 7 per group); **(C)** CD4 immunostaining of skin lesions from control and LL-37-induced mice (scale bar, 50 μm); **(D)** quantification of dermal CD4 fluorescence intensity in mice treated with PBS or Spe (n = 7 per group); **(E–G)** RT-qPCR analysis of TNF-α, IL-6, and IL-1β expression; and **(H–J)** ELISA analysis of TNF-α, IL-6, and IL-1β serum levels. Data are presented as mean ± SEM, *p < 0.05, **p < 0.01, ***p < 0.001. NS, not significant (p > 0.05).

Neurovascular dysregulation is thought to play an important role in rosacea pathogenesis (33). To assess cutaneous vasculature status, CD31 (a vascular marker) immunostaining was performed. LL-37-treated mice exhibited significantly increased mean CD31 fluorescence intensity (p < 0.001), indicating active angiogenesis. In contrast, Spe administration significantly reduced CD31 intensity in a dose-dependent manner (p < 0.01 or p < 0.001), reflecting suppression of skin angiogenesis. However, CD31 fluorescence intensity was significantly increased in the Spe 60 + KLK5 group compared with the Spe 60 group (p < 0.001), suggesting that KLK5 counteracted the inhibitory effect of Spe and confirming that Spe reduces angiogenesis in rosacea by targeting KLK5 (**Figures 4A, B**).

### 3.6 Spe inhibits CD4^+^ T-cell infiltration in LL-37-induced cutaneous inflammation

CD4^+^ T-cell infiltration suggests that adaptive immunity contributes to rosacea pathogenesis (34). Immunofluorescence analysis revealed markedly increased mean CD4^+^ T-cell fluorescence intensity in the model group compared with controls (p < 0.001). Spe treatment dose-dependently reduced CD4^+^ T-cell infiltration (p < 0.001), thereby ameliorating rosacea-like inflammation. Conversely, CD4^+^ T-cell infiltration was significantly elevated in the Spe 60 + KLK5 group compared with the Spe 60 group (p < 0.001) (**Figures 4C, D**).

Previous studies have shown that CD4^+^ T cells promote the secretion of pro-inflammatory cytokines such as TNF-α, IL-1β, and IL-6. Our findings demonstrate a correlation between CD4^+^ T-cell infiltration and elevated levels of these cytokines in lesional skin and serum, suggesting that these cells may contribute to the inflammatory milieu in this model (35). TNF-α regulates inflammation, immunity, homeostasis, and tumor development (36). IL-6 and IL-1β also contribute to angiogenesis and inflammation in the tumor microenvironment (37). RT-qPCR analysis showed that LL-37 treatment significantly upregulated TNF-α, IL-1β, and IL-6 mRNA levels (p < 0.001), whereas Spe treatment dose-dependently suppressed these cytokines (p < 0.05 or p < 0.001) (**Figures 4E–G**). In the present study, we investigated the therapeutic effects of Spe in the LL-37-induced rosacea-like mouse model. Serum ELISA confirmed these results, showing elevated TNF-α, IL-1β, and IL-6 in LL-37-treated mice (p < 0.001), which were reduced by Spe treatment in a dose-dependent manner (**Figures 4H–J**). In the Spe 60 + KLK5 group, cytokine expression in both skin and serum was significantly increased compared with the Spe 60 group (p < 0.001). These findings demonstrate that Spe suppresses LL-37-induced CD4^+^ T-cell infiltration and cytokine production, thereby modulating adaptive immune responses in rosacea.

### 3.7 Spe attenuates inflammation in rosacea-like mice by inhibiting KLK5

**Figure 5:**
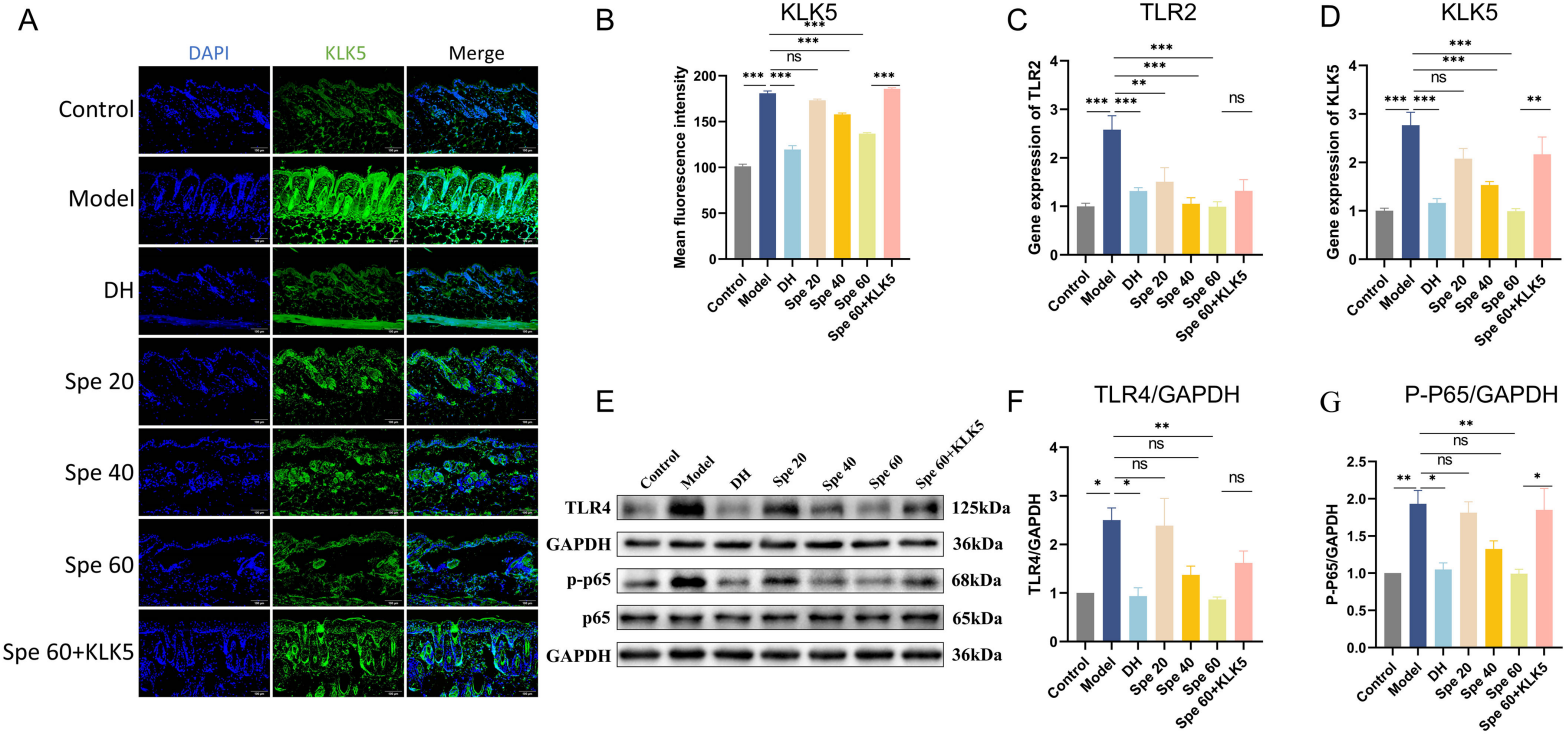
Effects of Spe on KLK5 and TLR4/NF-κB pathway activity in rosacea-like mouse skin. **(A)** Immunostaining of KLK5 in skin lesions from control and LL-37-induced mice (scale bar, 50 μm); **(B)** quantification of dermal KLK5 fluorescence intensity (n = 7 per group); **(C–D)** TLR2 and KLK5 expression levels by RT-qPCR; **(E)** Western blot analysis of TLR4, p-p65, and GAPDH; **(F)** ratio of TLR4/GAPDH (n = 3); and **(G)** ratio of p-p65/GAPDH (n = 3). Data are presented as mean ± SEM, *p < 0.05, **p < 0.01, ***p < 0.001. NS, not significant (p > 0.05).

To verify the inhibitory effect of Spe on KLK5, KLK5 protein expression was examined by immunofluorescence staining. As shown in **Figures 5A, B**, the model group exhibited a significant increase in average fluorescence intensity of KLK5 protein compared with the control group (p < 0.001). In contrast, both Spe and DH treatments suppressed KLK5 expression (p < 0.001), with Spe producing a dose-dependent effect. To further confirm these observations, RT-qPCR was performed to evaluate KLK5 expression, and the results were consistent with immunofluorescence findings, demonstrating that Spe treatment significantly reduced KLK5 expression in rosacea-like mice in a dose-dependent manner (p < 0.001 or NS).

Toll-like receptors (TLRs) are pattern recognition receptors that play key roles in immune and inflammatory responses and are broadly expressed on the membranes of macrophages, dendritic cells, and lymphocytes (38). Epidermal TLR2 expression has been shown to be elevated in patients with rosacea compared with healthy controls. This increased TLR2 activity in keratinocytes results in elevated KLK5 expression (39). The upregulation of TLR2 has been linked to aberrant KLK5 activity, altered LL-37 levels, and induction of rosacea symptoms (40). RT-qPCR analysis of TLR2 revealed that Spe treatment significantly reduced TLR2 expression in a dose-dependent manner (p < 0.001 or p < 0.01), suggesting that Spe inhibits KLK5 expression partly by downregulating TLR2 (**Figure 5C**). To further verify the inhibitory effect on KLK5, KLK5 was injected subcutaneously into the Spe 60 group of mice. Immunofluorescence staining and RT-qPCR results showed a significant increase in KLK5 protein and gene expression (p < 0.05), indicating that the elevated expression of KLK5 reversed the therapeutic effect of high-dose Spe in rosacea-like mice (**Figures 5B, D**). In conclusion, the results demonstrate that Spe attenuates rosacea-like inflammation by inhibiting KLK5 expression.

### 3.8 Spe inhibits activation of the TLR4/NF-κB pathway in rosacea-like mice

TLR4 is known to exacerbate inflammatory responses through activation of the NF-κB pathway and upregulation of inflammatory mediators (41). Therefore, the effect of Spe on the TLR4/NF-κB pathway was examined. As shown in **Figures 5E–G**, the LL-37-induced rosacea mouse model demonstrated a significant increase in TLR4 and phosphorylated p65 (p-p65) expression (p < 0.05 or p < 0.01). Conversely, Spe treatment markedly reduced both TLR4 expression and NF-κB phosphorylation in a dose-dependent manner. The Spe 60 + KLK5 group exhibited significantly higher TLR4 and p-p65 expression compared with the Spe 60 group (p < 0.05 or NS), indicating that KLK5 reactivation counteracted Spe-mediated suppression. These results confirm that Spe inhibits KLK5 to suppress the TLR4/NF-κB signaling pathway.

### 3.9 Spe modulates serum metabolomics in rosacea mice

To investigate the effect of Spe on metabolic pathways, an untargeted metabolomics approach was employed. Separation analysis among experimental groups was conducted using log-transformed raw data, followed by one-way statistical testing (t-test or Wilcoxon test) and multifactorial analysis with orthogonal partial least squares-discriminant analysis (OPLS-DA) using the R package ropls. Differential metabolites were screened using thresholds of p < 0.05 and VIP > 1.0.

**Figure 6:**
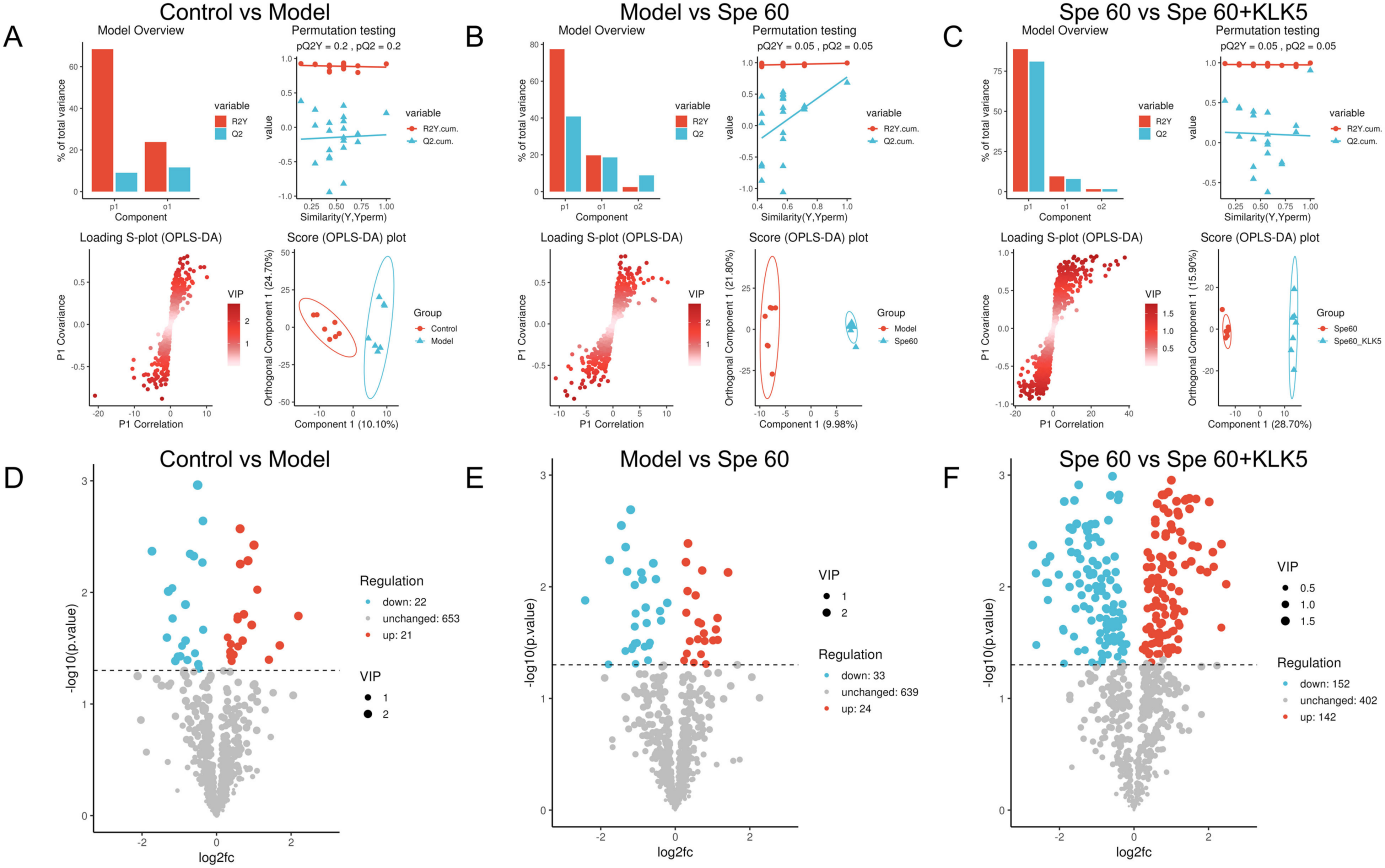
Effects of Spe on serum metabolomics in rosacea-like mice. **(A–C)** OPLS-DA analysis; and **(D–F)** Volcano plots of differential metabolites. Data are presented as mean ± SEM, *p < 0.05, **p < 0.01.

OPLS-DA analysis revealed clear separation between the control and model groups (R2X = 0.347, R2Y = 0.922, Q2 = 0.206), between the model and Spe 60 groups (R2X = 0.429, R2Y = 0.995, Q2 = 0.682), and between the Spe 60 and Spe 60 + KLK5 groups (R2X = 0.526, R2Y = 0.997, Q2 = 0.904), suggesting significant metabolic differences across groups (**Figures 6A–C**). Volcano plots further illustrated the distribution of differential metabolites (**Figure 6D**).

A total of 43 differential metabolites were identified between the control and model groups, with 21 upregulated in the model group (e.g., ethylmalonic acid, isobutyrylglycine, acetoin, epigallocatechin, trimethylamine N-oxide, p < 0.01; LPE 20:2_sn1, p < 0.05) and 22 downregulated (e.g., indoleacrylic acid, loliolide, Gly-Phe, pyroglu-Val, tridecanedioic acid, tetradecanedioate (C14-DC), FA 18:2 + 3O riboflavin, gamma-caprolactone, p < 0.01). Importantly, 26 of these metabolites were restored toward normal levels by Spe treatment.

Between the model and Spe 60 groups, 57 differential metabolites were identified (**Figure 6E**), with 24 upregulated in the Spe 60 group (e.g., 3-indoleacetonitrile, L-methionine sulfone, pyroglu-Val, Asp-Glu, PC 38:5|PC 18:1_20:4, PC 40:7|PC 18:1_22:6, p < 0.01) and 33 downregulated (e.g., 2-hydroxycaproic acid, phenyllactic acid, Gly-Val, L-arginine, isoferulic acid, lauryl sulfate, LPE 15:0, LPA 20:4_sn1, LPS 16:0, LPE 20:2_sn1, 2-hydroxyisovaleric acid, p < 0.01). Between the Spe 60 and Spe 60 + KLK5 groups, 294 differential metabolites were identified (**Figure 6F**), including 152 upregulated in the Spe 60 group (e.g., 3-hydroxy-2-ethylpropionate, glycolic acid, LPE O-20:4, LPC 16:1_sn1, eriodictyol, p < 0.01) and 142 downregulated (e.g., PE 34:2|PE 16:0_18:2, LPE 15:0, LPS 16:0, alpha-hydroxyisocaproate, P < 0.01). Among these, unsaturated fatty acids were predominant.

**Figure 7:**
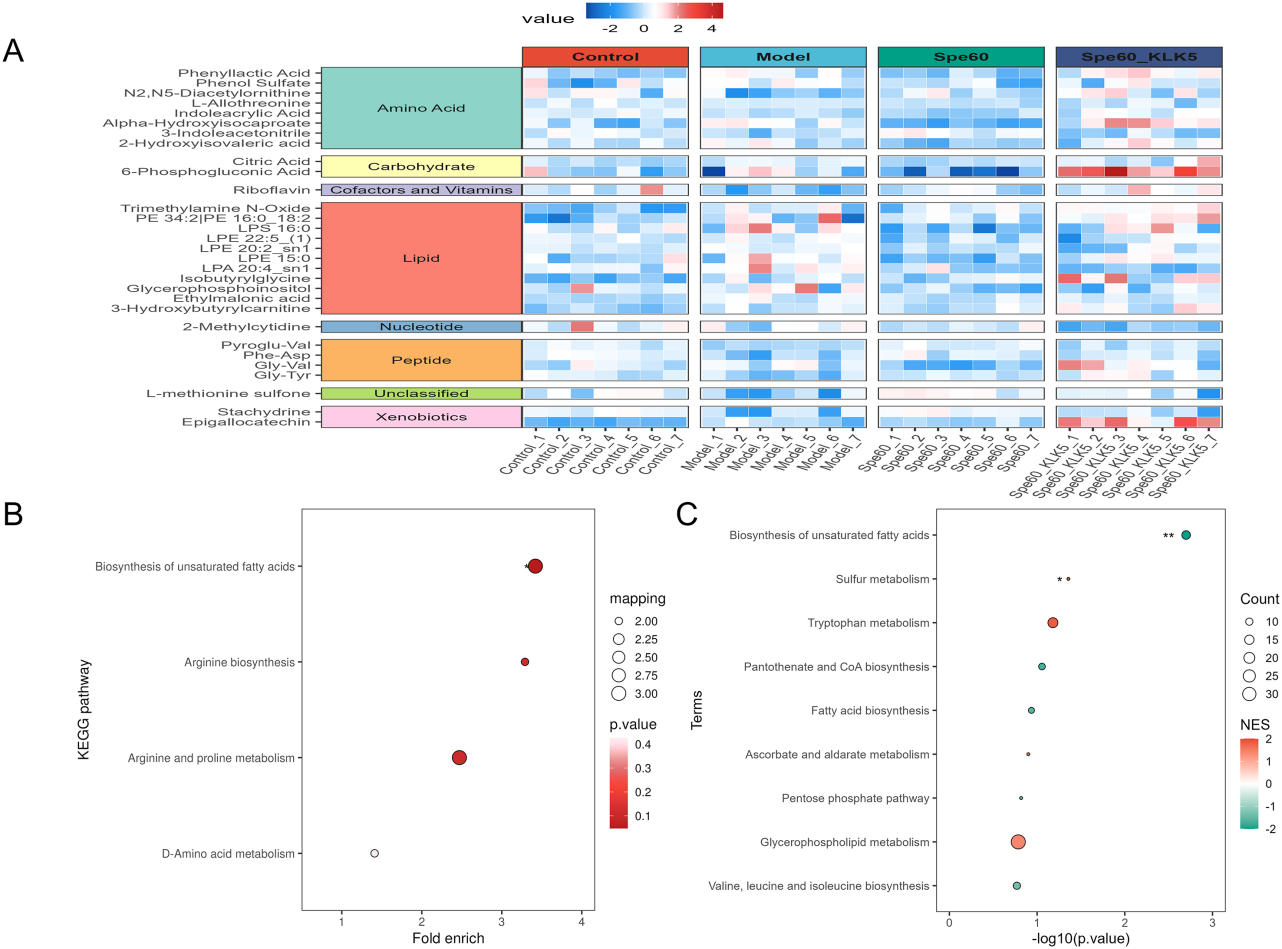
Effects of Spe on serum metabolomics in rosacea-like mice. **(A)** Heat map of differential metabolites; **(B)** KEGG pathway enrichment analysis; and **(C)** MSEA analysis.

A heat map generated from univariate and multivariate analyses (**Figure 7A**) identified 30 key differential metabolites, primarily unsaturated fatty acids (e.g., LPE 20:2_sn1, LPA 20:4_sn1), that were closely related to lipid metabolism. Fisher’s exact test was then applied to evaluate pathway enrichment, revealing significant enrichment in biosynthesis of unsaturated fatty acids, arginine biosynthesis, arginine and proline metabolism, and D-amino acid metabolism (**Figure 7B**).

Furthermore, MSEA based on all identified metabolites and KEGG pathways demonstrated enrichment in unsaturated fatty acid biosynthesis, sulfur metabolism, tryptophan metabolism, and glycerophospholipid metabolism (**Figure 7C**). Together, KEGG and MSEA results strongly suggest that Spe ameliorates rosacea by regulating unsaturated fatty acid biosynthesis.

## 4 Discussion

Rosacea is a widespread chronic inflammatory skin disease that can cause disfigurement, depression, and reduced quality of life (42). Previous research suggests that immune system activation and neurovascular dysregulation may be two of the pathophysiological processes of rosacea (43). Traditional therapies for rosacea, including antibiotics and vitamin A analogs, have not produced satisfactory outcomes (44). Spe, a secoiridoid found in *Ligustri Lucidi Fructus*, has been shown to prevent carbon tetrachloride-induced liver injury in rats by reducing oxidative stress through the activation of nuclear factor erythroid 2-related factor 2 signaling and by decreasing hepatocyte apoptosis (45). Furthermore, Spe reduced IL-1β, IL-6, and TNF-α levels in diabetic nephropathy rats (16). However, current literature does not demonstrate the effect of Spe on rosacea.

Molecular docking combined with virtual screening (VS) has rapidly become an important method for drug discovery and development (46). In this study, a comprehensive structure-based VS approach was used to identify Spe as an efficient and selective KLK5 inhibitor. The binding interaction assessment revealed that Spe exhibited a binding affinity of –117.28 kcal/mol for KLK5. This indicates that Spe can effectively contribute to KLK5 inhibitory functions. It is noteworthy that molecular dynamics simulations of the KLK5-Spe complex further revealed highly stable interaction characteristics. These results highlight the potential of Spe as a lead compound for developing therapeutic strategies for rosacea.

Spe is a key component in many herbal preparations and has considerable anti-inflammatory properties (15). In the present study, Spe treatment improved the LL-37-induced rosacea-like phenotype by reducing the inflammatory response. The protective effect of Spe was confirmed by evaluating the infiltration of inflammatory cells into the dermis. The findings indicate that Spe reduced inflammatory cell infiltration in the skin of rosacea-like mice. Abnormalities in the innate immune system and neurovascular dysregulation are considered the primary causes of rosacea (47). The innate immune system includes Toll-like receptor (TLR) families, which detect external environmental stimuli such as UV radiation, microorganisms, and chemical or physical damage. Activation of the innate immune system causes an increase in cytokine and antimicrobial compound production in the skin (48). In rosacea, environmental stimuli that activate TLR2 enhance KLK5 expression, leading to the accumulation of LL-37, which plays a major role in disease pathogenesis. Azelaic acid is an effective rosacea therapy because it suppresses innate immune responses (49). In this study, Spe was shown to substantially inhibit LL-37-induced TLR2 expression.

To further investigate the inhibitory effect of Spe on KLK5-mediated inflammation, KLK5 was administered subcutaneously into the dorsal surface of mice. KLK5 injection induces LL-37 expression through proteolytic cleavage of human cationic antimicrobial protein 18 (HCAP18). Spe treatment reduced KLK5 protein activity. In the Spe 60 group, subcutaneous KLK5 injection caused erythema and exacerbated skin inflammation. This indicates that Spe suppresses KLK5-driven inflammation by inhibiting KLK5 protein activity. Meanwhile, TLR2 stimulation of adaptive and innate immune responses is another pathogenic mechanism of rosacea. TLR2 activation stimulates mast cells by increasing LL-37 synthesis (43). Current *in vivo* approaches primarily reflect pharmacological interventions targeting downstream inflammatory cascades rather than capturing the complete physiological dynamics of endogenous KLK5 expression, processing, and activation. Recent studies have shown that mast cells are critical mediators of LL-37-induced inflammation in rosacea and that matrix metallopeptidase 9 (MMP-9), a key marker of mast cell activation, plays an important role in this process (32). The present study demonstrated that Spe significantly reduced mast cell counts and LL-37-induced MMP-9 expression in the skin.

Besides inflammatory hyperactivation, rosacea is also characterized by vascular system dysfunction (50). Rosacea symptoms, such as flushing and burning sensations, are attributed to neurovascular pathology (51). CD31, a marker of vascular endothelial cells, is widely used to detect and quantify neovascularization (42). In rosacea-like mice, Spe reduced angiogenesis and markedly lowered CD31 expression in lesions. Although evidence on Spe’s anti-angiogenic activity is limited, these findings suggest that Spe can suppress angiogenesis in rosacea-like conditions. However, further studies are required to elucidate the mechanisms of this anti-angiogenic effect.

CD4^+^ T cells play a role in activating immune responses by differentiating into T helper (Th) effector cells (52). Elevated CD4^+^ T cells, along with increased Th1 and Th17 cytokines, have been observed in rosacea lesions, indicating hyperactivation of the adaptive immune system (53). In this study, Spe reduced LL-37-induced infiltration of CD4^+^ T cells.

The Toll-like receptor 4 (TLR4) pathway represents a promising strategy for treating a variety of illnesses, including atherosclerosis, rheumatoid arthritis, neuroinflammation, trauma, and hemorrhage (54). NF-κB, a nuclear transcription factor, promotes inflammation through classical and alternative mechanisms. Inactive NF-κB remains in the cytoplasm (55, 56). Upon phosphorylation, NF-κB is activated and translocates into the nucleus, inducing the production of inflammatory cytokines such as TNF-α, IL-1β, and IL-6, which contribute to tissue damage and inflammation (57). Overproduction of these cytokines has been linked to inflammatory disorders (58). Lipopolysaccharides (LPS) bind to TLR4 and activate NF-κB, leading to transcription of pro-inflammatory cytokines (59). This study provides the first evidence that Spe therapy alleviates rosacea by blocking the KLK5 and TLR4/NF-κB pathways.

Metabolomics, a powerful tool for large-scale identification of metabolites, has proven highly effective in studying physiological states, identifying biomarkers, mapping disrupted pathways, and diagnosing diseases (60). Serum metabolic profiling is particularly promising for disease biomarker discovery (61). In this study, untargeted metabolomics identified 30 potential biomarkers associated with LL-37-induced rosacea before and after Spe treatment. The main metabolic pathways involved in rosacea pathogenesis were linked to unsaturated fatty acid biosynthesis, arginine metabolism, proline metabolism, and D-amino acid metabolism. Derivatives of unsaturated fatty acids function as anti-inflammatory mediators that suppress cytokine production in LPS-stimulated macrophages (62). The arginase (ARG) pathway, which is crucial for cellular function, is disrupted in conditions such as Alzheimer’s disease, psoriasis, and delayed wound healing. Epidermal ARG regulates keratinocyte responses during wound repair (63). Arginine metabolites, such as proline, nitric oxide (NO), and polyamines, influence every stage of the wound healing process (64). D-amino acids have been linked to bacterial growth, biofilm formation, diffusion, and peptidoglycan metabolism (65). These results suggest that Spe modulates multiple metabolic pathways to alleviate rosacea. Following LL-37 induction, increased levels of LPE 20:2_sn1 and LPA 20:4_sn1 were observed, which decreased after Spe therapy. This supports the hypothesis that Spe may mitigate rosacea progression by regulating unsaturated fatty acid biosynthesis. While the serum metabolomic data presented here reflect distal systemic consequences rather than the local drivers of lesional pathology, and the observed metabolite–pathway associations remain only statistical correlations, further targeted functional validation will be essential to establish their causal relevance and underlying mechanistic roles.

## 5 Conclusion

In summary, this study identified Spe as a KLK5 inhibitor through virtual screening and suggested that it may be a promising candidate for the treatment of rosacea, although further validation is still required. Intraperitoneal Spe treatment markedly improved LL-37-induced rosacea-like symptoms, including erythema, elevated inflammatory cytokines, mast cell infiltration, cutaneous angiogenesis, and immune cell infiltration. Serum metabolomics and animal experiment results suggest that Spe directly binds to KLK5 and inhibits its expression and activity, thereby blocking the downstream LL-37/TLR4/NF-κB inflammatory cascade. In addition, Spe treatment was associated with metabolic remodeling, particularly involving unsaturated fatty acid biosynthesis, which may further contribute to its anti-inflammatory effects. These findings establish KLK5 inhibition as the central mechanism underlying the protective effects of Spe in rosacea-like dermatitis. This is the first study to elucidate the therapeutic mechanism of Spe in rosacea, supporting its potential as a natural therapeutic agent and warranting further investigation.

## Data Availability

The original contributions presented in the study are included in the article/supplementary material. Further inquiries can be directed to the corresponding authors.
